# Septic pericarditis and pneumopericardium in a dog with an oesophageal foreign body

**DOI:** 10.4102/jsava.v88i0.1496

**Published:** 2017-05-29

**Authors:** Willem J. Botha, Varaidzo Mukorera, Robert M. Kirberger

**Affiliations:** 1 Department of Companion Animal Clinical Studies, University of Pretoria, South Africa

## Abstract

A 5-year-old, intact, male Yorkshire Terrier presented with a 6-day history of lethargy and anorexia. Clinical examination revealed dental plaque accumulation, abdominal effort during respiration and muffled heart sounds. Thoracic radiographs revealed an enlarged globoid cardiac silhouette and mild pneumopericardium, transthoracic ultrasonography revealed a pericardial effusion after which pericardiocentesis, cytology and culture diagnosed septic pericarditis. Three multidrug-resistant bacteria were isolated, two of which have been implicated in gas-producing infections before. Medical management failed to resolve the pericarditis and euthanasia was opted for. A chronic osseocartilaginous oesophageal foreign body cranial to the heart base was found on necropsy. Septic pericarditis and pneumopericardium are rare conditions in dogs. This is the first case to describe a multidrug-resistant polybacterial aetiology causing mild pneumopericardium and only the second case to describe septic pericarditis associated with an oesophageal foreign body.

## Introduction

Neoplastic and idiopathic exudates compose the majority of pericardial effusions in dogs (Berg & Wingfield 1984; Johnson et al. 2004). Septic pericarditis causing pericardial effusion is rare (Johnson et al. 2004). Fungi (aspergillosis, *Candida albicans* and coccidioidomycosis), bacteria (*Acinetobacter* spp., *Actinomyces, Bacteroides, Nocardia, Pasteurella multocida, Pseudomonas, Staphylococcus, Streptococcus* and tuberculosis) and parasites (dirofilariasis and leishmaniasis) have been implicated in septic pericarditis (Casamián-Sorrosal et al. 2008; Majoy et al. 2013; Mohri et al. 2009; Peterson et al. 2003). Septic pericarditis is typically associated with an inciting cause such as bite wounds (Fuentes et al. 1991), intrapericardial foreign body, migrating grass awns (Aronson & Gregory 1995; Straw, Ogburn & Wilson 1979), administration of immunosuppressive drugs (Mohri et al. 2009), haematogenous spread (Lobetti 2007; Peterson et al. 2003) and local extension of infection (Miller, Fox & Saunders 2004). Septic pericarditis treatment comprises pericardiocentesis with drainage and administration of appropriate antimicrobials with pericardiectomy indicated in cases needing continued drainage (Aronson & Gregory 1995).

This report describes a case of multidrug-resistant, polybacterial septic pericarditis with associated pneumopericardium in a Yorkshire Terrier with a chronic, subclinical oesophageal foreign body.

## Ethical considerations

This is a retrospective case report describing a case of a client-owned pet using data retrieved and reviewed from clinical records of the Onderstepoort Veterinary Academic Hospital. The pet, while alive, was treated and housed according to standard hospital protocols for management of client-owned pets. No tests or treatments were conducted for research purposes. The owner provided standard signed consent for the data and images to be used for publication.

## Case history

A 5-year-old, intact, male Yorkshire Terrier was referred with complaints of lethargy and inappetence of 6 days’ duration that was unresponsive to a variety of antibiotics (cephalexin, metronidazole, enrofloxacin and amoxicillin clavulanate; dosages, duration of treatment and manufacturers not recorded). The dog came as the only pet from a suburban residential property with no history of travel or previous medical conditions. On presentation, the dog was in a poor body condition (2/5) with severe dental plaque formation, with associated periodontitis and an abdominal component to respiration. The heart sounds were muffled, mucous membranes were pale pink, capillary refill time was 1.5 s and peripheral pulses were of normal quality.

Urine collected via cystocentesis was dark yellow and cloudy, adequately concentrated, with mild proteinuria on dipstick examination. Routine haematology and serum biochemistry indicated a severe left shift neutrophilia, moderate hypoalbuminaemia and mild hypokalaemia (Table 1).

**TABLE 1:** Haematology and serum biochemistry abnormalities.

Test	Result	Unit	Reference interval
White cell count	35.17	× 10^9^/L	6–15
Segmented neutrophils	27.43	× 10^9^/L	3–11.5
Band neutrophils	6.33	× 10^9^/L	0–0.5
Lymphocyte	0.70	× 10^9^/L	1–4.8
Monocyte	0.00	× 10^9^/L	0.15–1.35
Eosinophil	0.00	× 10^9^/L	0.1–1.25
Albumin	14.20	g/L	28–41
Potassium	3.00	mmol/L	3.6–5.1

**FIGURE 1:**
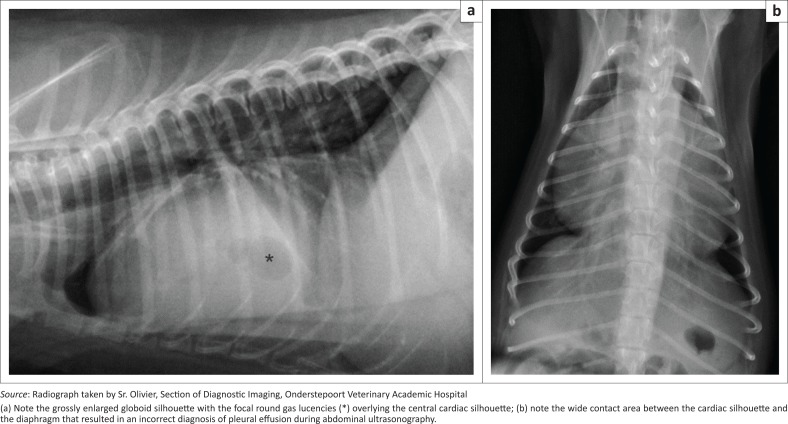
Right lateral (a) and DV (b) thoracic radiographs.

**FIGURE 2:**
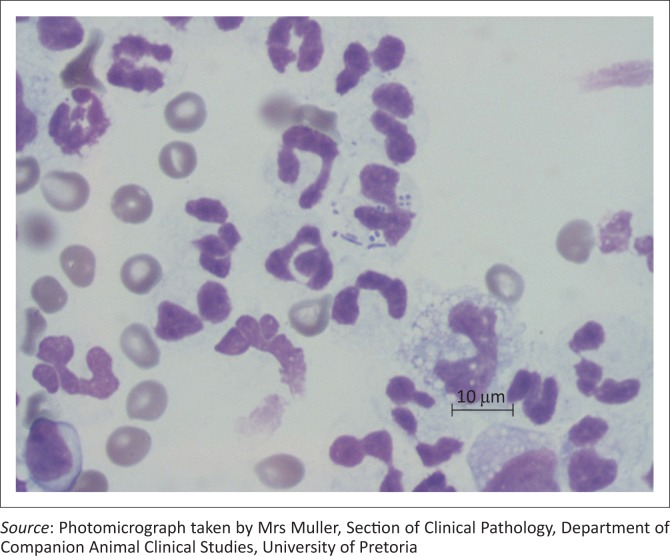
Light microphotographs of the pericardial effusion smears. There is a predominance of degenerate neutrophils with phagocytosing rods and cocci. Three species of bacteria were isolated from the pericardial fluid.

Abdominal ultrasonography revealed incidental small renoliths and a cursory examination of the thoracic cavity using a sub-xiphoid transhepatic window revealed the absence of a normal mirror image artefact because of the presence of anechoic fluid with multiple echogenic specks, many of which had distal reverberation artefacts. An ultrasonographic diagnosis of suspected pleural effusion with intermingled free gas was made. Further examinations then focussed on the thorax. Orthogonal thoracic radiographs revealed a grossly enlarged, globoid cardiac silhouette occupying six intercostal spaces with a large area of cardiodiaphragmatic contact of up to seven sternebrae (Figure 1). On the lateral view within the cardiac silhouette, there were at least three central gas lucencies, the largest being 12 mm in diameter. There was no evidence of pulmonary or pleural pathology. A differential diagnosis of peritoneal-pericardial diaphragmatic hernia (PPDH) was considered but the lack of other radiographic changes indicative of PPDH, such as the presence of intestinal bowel loops within in the cardiac silhouette, made this unlikely and the presence of free gas, possibly produced by bacteria in the pericardium, was suspected. The presence of free gas in the pericardial effusion was confirmed by transthoracic ultrasonography. The pericardial fluid was up to 12 mm wide and resulted in minor right atrial wall tamponade. Pericardiocentesis and drainage yielded 25 mL of turbid, red-yellow fluid with the width of effusion being reduced to 2 mm immediately post drainage. Fluid analysis confirmed the presence of a septic exudate. Cytology revealed a predominance of degenerate neutrophils phagocytosing rods and two morphologically distinct cocci suggesting a polybacterial infection (Figure 2). Culture and antibiogram of the septic exudate isolated *Enterococcus faecalis, Klebsiella pneumoniae* and methicillin-resistant *Staphylococcus aureus* (MRSA). This corresponded with the cytological findings of a bacillus shaped colony (*Klebsiella pneumoniae*) and two coccoid colonies (*Enterococcus faecalis* and *Staphylococcus* spp.) seen. All the isolates were extensively multidrug-resistant to antibiotics routinely used in susceptibility testing. A diagnosis of septic pericarditis with associated pneumopericardium was made.

**FIGURE 3:**
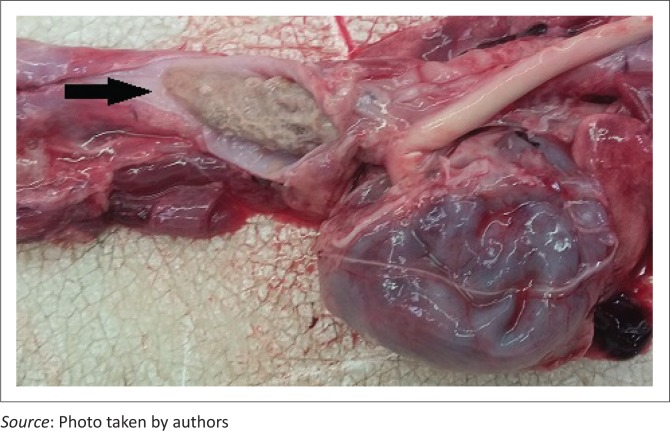
Post mortem photograph. Note the cartilaginous foreign body, indicated by the arrow, clogged with food lodged in the oesophagus cranial to the heart base.

**FIGURE 4:**
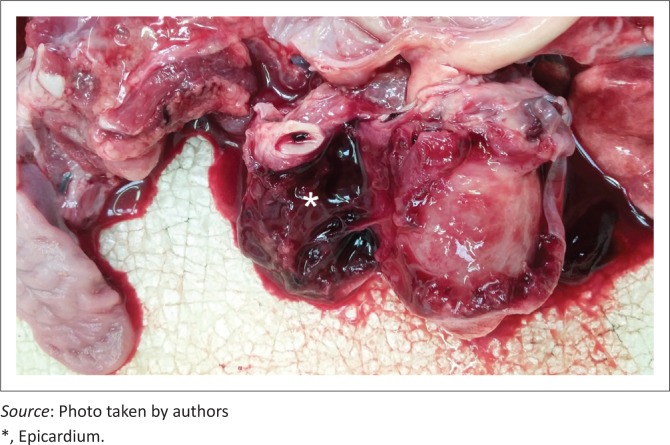
Post mortem photograph. The pericardial sac is transected and reflected to expose the epicardium. Note the severe fibrous changes associated with the epicardial surfaces and pericardial sac consistent with a constrictive pericarditis.

The owner declined pericardiectomy because of financial constraints and medical management was pursued. Prior to receiving the antibiogram results, the dog was treated with amoxicillin clavulanic acid (Sandoz Co-amoxyclav 0.6 g, Sandoz SA [Pty] Ltd) at 20 mg/kg three times daily intravenously and enrofloxacin (Baytril 5%, Bayer [Pty] Ltd) at 5 mg/kg once daily subcutaneously. The enrofloxacin was later replaced with amikacin (Amikacin-Fresenius, Fresenius Kabi) at 20 mg/kg once daily intravenously following the antimicrobial susceptibility findings. Intravenous lactated Ringer’s solution (Ringer-lactate solution, Fresenius Kabi) was given at 7 mL/hr and supplemented with 40 mmol potassium chloride (Adco-Potassium Chloride 15%, Adcock Ingram Ltd). Five days after the initial diagnosis, follow-up thoracic ultrasonography showed a very mild increase in pericardial effusion with the formation of some fibrin tags but no indication of cardiac tamponade. Eight days after admission, the dog developed a moderate modified transudate ascites and thoracic ultrasonography revealed worsening of the pericardial effusion (5 mm wide, right parasternal short axis view, sternal recumbency) with extensive echogenic fibrin tags accompanied by right atrial and ventricular wall fluttering indicative of cardiac tamponade, probably as a result of constrictive pericarditis. The owner opted for euthanasia because of financial constraints and a poor prognosis. Necropsy confirmed a septic, constrictive pericarditis with associated congestive, right-sided heart failure. In addition, a piece of cartilage (5 cm × 2 cm) was present in the oesophagus cranial to the heart base that was associated with local chronic ulcerative oesophagitis (Figures 3 and 4). No communication between the oesophagus and pericardial sac could be demonstrated.

## Discussion

Pericardial effusion, including septic pericarditis, mostly affects large breed dogs (Johnson et al. 2004). In one case report of septic pericarditis in a Yorkshire Terrier, the underlying aetiology was unknown (Wagner et al. 2006). In our case, the exact underlying aetiology is speculative.

Haematogenous spread secondary to periodontitis was considered a plausible aetiology and has been suggested as the underlying aetiology in a cat with periodontitis after a dental procedure (Lobetti 2007). However, this was deemed unlikely in our case considering the polybacterial nature of the pericarditis.

Polybacterial infections are commonly reported in septic pericarditis cases (Aronson & Gregory 1995; Stafford Johnson, Martin & Stidworthy 2003) and are suggestive of direct inoculation, opportunistic colonisation or secondary to immunocompromise (Wagner et al. 2006). Initial oesophageal-pericardial penetration that healed again could have been the source of infection or migrating gas in our case. A penetrating osseous oesophageal foreign body causing septic pericarditis has been reported once before (Kolm et al. 2001). Survey thoracic radiographs revealed no signs of the oesophageal osseocartilaginous foreign body on initial assessment. There was, thus, no indication to perform more advanced imaging techniques or endoscopy to find a foreign body. Careful retrospective review of the radiographs with the necropsy findings in mind facilitated identification of a possible oesophageal foreign body at the thoracic inlet. Finally, the lack of a visible communication and mediastinitis on necropsy makes traumatic penetration into the pericardium questionable.

Initially, the pericardial effusion was mistakenly diagnosed as pleural effusion on abdominal ultrasonography because of the wide contact area between the pericardium and the diaphragm. However, the septic nature of the fluid was correctly identified because of the presence of reverberating echogenic specks in the fluid indicative of minute gas bubbles. Radiologically, at least three gas bubbles were seen in the uppermost part of the cardiac silhouette enabling a diagnosis of pneumopericardium. Pneumopericardium is defined as air accumulation within the pericardial cavity and rarely reported in the dog and then usually without pericardial fluid (Maki et al. 1999). Experimentally induced pneumopericardium in the dog has been shown to cause similar haemodynamic compromise to pericardial effusion resulting in cardiac tamponade (Reed & Thomas 1984). Pneumopericardium secondary to trauma, pneumonia, pneumomediastinum, lung abscess, bronchopulmonary disease and pulmonary-pericardial communication has been reported but was of a more severe nature compared with the mild pneumopericardium seen in our case (Borgonovo et al. 2014; Hassan, Torad & Shamaa 2015). Gas entering during an oesophageal-pericardial penetration would have been resorbed over time and, therefore, it is considered more likely that the intrapericardial bacteria were the source of active gas production. *Staphylococcus aureus* and *Klebsiella pneumoniae* have been implicated in gas-producing tissue infections in humans (Lee et al. 2014; Saliba et al. 2013).

Literature on the management of bacterial septic pericarditis is scarce. Pericardiectomy has been shown to be effective in the management of septic pericarditis and a prolonged median survival time has been reported with idiopathic pericardial effusion cases treated with pericardiectomy (Aronson & Gregory 1995; Casamián-Sorrosal et al. 2008; Gilson et al. 2005; Johnson et al. 2014; Veloso et al. 2014). However, pericardiectomy appears necessary in most cases to prevent restrictive pericarditis and, if not done, is associated with a poor prognosis (Aronson & Gregory 1995; Kolm et al. 2001; Peterson et al. 2003; Stafford Johnson et al. 2003).

Cytology provides a definitive diagnosis in infectious pericarditis cases (Cagle et al. 2014). Culture may diagnose bacteria not identified on cytology (Aronson & Gregory 1995). The multidrug-resistant nature of the isolates in our case was concerning.

*Enterococcus faecalis* is a commensal in dog intestines and considered a leading nosocomial infection with extensive levels of multidrug resistance (Jackson et al. 2009). Community acquired MRSA is uncommon in pets but endemic in many human healthcare facilities (Weese et al. 2006). *Klebsiella pneumonia* is a gram-negative, facultatively anaerobic bacterium considered to be a minor intestinal commensal but commonly associated with opportunistic and nosocomial infections (Glickman 1981). Interspecies transmission of bacteria between dogs and humans has long been suspected but is difficult to confirm. As both the owners of the dog were clinical health professionals, they could have been exposed to, and possibly colonised with, multiple multidrug-resistant microorganisms because of chronic exposure. Because of the limited and brief contact periods in the referring hospital environment, community-acquired infections of multidrug-resistant bacteria seem more plausible. However, the multitude of antimicrobial agents used prior to referral conceivably contributed to the selection of the multidrug-resistant isolates. The multidrug-resistant nature of the infection emphasises the need for judicial antimicrobial usage in all aspects of veterinary practice.

## Conclusion

To the authors’ knowledge, this is only the second case report to describe septic pericarditis possibly associated with an oesophageal foreign body and the first case to describe pneumopericardium secondary to multidrug-resistant polybacterial septic pericarditis.
